# Urine analysis in monoclonal gammopathies at diagnosis: settling cut-off values

**DOI:** 10.1515/almed-2024-0045

**Published:** 2024-09-23

**Authors:** Mònica Vidal-Pla, Elisa Nuez-Zaragoza, Indira Bhambi, Vicente Aguadero

**Affiliations:** Biochemistry Department, Clinical Laboratory, 16382 Parc Taulí University Hospital, Institut d’Investigació i Innovació Parc Taulí I3PT, Universitat Autònoma de Barcelona, Sabadell, Barcelona, Spain; Laboratory Department, Sant Joan Despí Moisès Broggi Hospital, CLILAB Diagnòstics, Barcelona, Spain

**Keywords:** cut-off, monoclonal gammopathy, M-protein, urine analysis

## Abstract

**Objectives:**

Laboratory testing has an extensive role in the diagnosis of monoclonal gammopathies. Since the last updates of the International Myeloma Working Group (IMWG) guidelines for the diagnosis of monoclonal gammopathies, debate has arisen as to whether urine analysis remains relevant for the diagnosis of these entities.

**Methods:**

We carried out a retrospective study with data from 132 patients with a newly diagnosed serum M-protein. Patients were divided into two groups depending on the presence of M-protein in urine and different variables were recorded and statistically compared between groups.

**Results:**

The aim of the study was to find a serum M-protein cut-off value under which urine analysis could be avoided in the first laboratory diagnosis. The results show that when the concentration of serum M-protein is ≤3.5 g/L and eGFR is >30 mL/min/1.73 m^2^ (sensitivity (S): 100 %, specificity (Sp): 49 %, negative-predictive value (NPV): 100 %) the probability of finding M-protein in urine is negligible. Patients with alpha heavy chain have a higher probability of having M-protein in urine. Thus, when the heavy chain of the M-protein is gamma or mu, serum cut-off value can be raised up to ≤4.9 g/L (S: 97 %, Sp: 52 %, NPV: 98 %).

**Conclusions:**

Settling these two cut-off values when a new M-protein is discovered could avoid a significant number of urine analyses, optimizing laboratory and healthcare resources.

## Introduction

Laboratory testing has an extensive role in the diagnosis of monoclonal gammopathies. Since the last updates of the International Myeloma Working Group (IMWG) guidelines for the diagnosis of monoclonal gammopathies, debate has arisen as to whether urine analysis remains relevant for the diagnosis of these entities 1], [2], [3.

The 2014 IMWG guidelines only consider urine analysis to be significant in the diagnosis of amyloidosis, light-chain monoclonal gammopathy of undetermined significance and smouldering myeloma. The diagnosis criteria of monoclonal gammopathy of undetermined significance (MGUS) are: serum M-protein <30 g/L, clonal bone marrow plasma cells <10 % and absence of CRAB symptoms. Urine M-protein concentration is not included as a criteria and in this case, the presence of urine M-protein wouldn’t change the diagnosis but it is still highly requested by the clinicians [1].

A 24 h urine is recommended for urine analysis, which is challenging for the patient to collect, rendering the analysis results inconsistent. Urine protein electrophoresis (UPE) and urine immunofixation electrophoresis (u-IFE) are time-consuming techniques that require a concentration step, and they are not fully automated. Furthermore, u-IFE requires a manual and expert interpretation [4, 5].

For these reasons we planned a study with the hypothesis that, when the serum M-protein concentration of a patient is low in the first laboratory analysis of a gammopathy, the probability of finding M-protein in urine is also low. The main aim of our study was to find a serum M-protein cut-off value under which the probability of finding a urine M-protein was very low and, hence, urine analysis could be avoided. We also evaluated whether other parameters help to predict the absence of M-protein in urine.

## Materials and methods

We performed a retrospective study in which patients with a newly discovered serum M-proteins were enrolled. Patients with biclonal paraproteins, monoclonal light chain paraproteins and amyloidosis were excluded. Secondary inclusion criteria comprised patients who had a u-IFE analysis within a maximum of two months after the discovery of the serum M-protein.

This observational retrospective study was approved by the Hospital’s Drug Research Ethics Committee (Code 20235011).

Our study population consisted of patients admitted in the hospital or derived from the outpatient care. Patient’s clinical data were collected by the hospital information management system to assure they met the inclusion criteria. The data recorded for each patient included age, sex, serum M-protein concentration (g/L), type of heavy chain (IgG, IgA, IgM), type of light chain (kappa or lambda) and total serum concentrations of the three immunoglobulins (IgG, IgA and IgM). We also recorded whether M-protein was present in urine, the total protein concentration in urine, and the estimation of glomerular filtration rate (eGFR), calculated by Chronic Kidney Disease Epidemiology Collaboration 2009 (CKD-EPI) equation. The patients were divided into one of two groups, depending on the presence of M-protein in urine: group A, patients without M-protein in urine; group B: patients with M-protein in urine (both intact monoclonal immunoglobulins and monoclonal light chain where considered as a positive result).

Serum proteinogram was performed by capillary electrophoresis using V8^®^ Nexus (Helena Biosciences Europe, Gateshead, UK). UPE was performed by gel electrophoresis using SAS3 (Helena Biosciences Europe, Gateshead, UK). Spikes observed on the serum and urine proteinograms were tested by immunofixation on SAS3 for confirmation and characterization. u-IFE were performed in all urine samples following an UPE. UV spectrometry was used to measure serum M-protein and densitometry to measure urine M-protein. In both cases a perpendicular method was used for integration.

Immunoglobulins were mesured on Optilite^®^ analyser (The Binding Site Group Ltd., Birmingham, UK) by turbidimetry. Proteins in urine were analysed by turbidimetry using Cobas 8000 on the c702 module (Roche Diagnostics, Mannheim, Germany).

Statistical analysis was performed using SPSS Statistics version 25.0 (IBM; Armonk, NY, USA), and statistical significance was set at p<0.05. Kolmogorov–Smirnov test was used to confirm the normal distribution of quantitative variables. To compare frequencies between groups A and B, we used chi-square tests or Fisher’s exact test. In order to compare the means of the quantitative variables between groups A and B, the Mann–Whitney U test was used. When the difference between the means of the two groups were statistically significant, we elaborated receiver operating characteristic (ROC) curves with the presence of M-protein in urine as the state variable.

## Results

Data from 132 patients corresponding to 132 samples were recorded between February 2019 and March 2022 (Tables 1 and 2). After analysing the data, a statistically significant and directly proportional relationship was found between the presence of urine M-protein and both, serum M-protein and urine total protein concentrations. Instead, the relationship is inversely proportional to eGFR and serum IgM concentrations. No statistically significant differences were found in the distribution of the patient’s age, and total serum concentration of IgG and IgA (Table 1).

**Table 1:** Patient demographics and clinical characteristics.

	Group A	Group B	p-Value	Area under the ROC curve (95 % CI)
	Median (IQR)	Median (IQR)		
Age, years	71 (20)	75 (20)	0.272	^a^
Serum M-protein, g/L	3.28 (4.51)	15.1 (29.1)	**<0.001**	0.794 (0.706–0.883)
TP in urine, g/L	0.10 (0.14)	0.45 (1.05)	**<0.001**	0.761 (0.663–0.857)
eGFR, mL/min/1.73 m^2^	78 (26)	59 (54)	**0.001**	0.691 (0.585–0.797)
IgG, g/L	12.22 (6.08)	11.69 (19.84)	0.516	^a^
IgA, g/L	2.54 (2.28)	1.39 (5.99)	0.088	^a^
IgM, g/L	0.995 (1.08)	0.32 (0.81)	**<0.001**	0.727 (0.609–0.845)

Statistically significant p-values are highlighted in bold. ^a^No ROC curve was elaborated as the variable was not statistically significant. CI, confidence interval; eGFR, estimated Glomerular Filtration Rate; IgA, immunoglobulin A; IgG, immunoglobulin G; IgM, immunoglobulin M; IQR, interquartile range; ROC, receiver operating characteristic; TP, total protein.

**Table 2:** Patient distribution according to sex and type of heavy and light chain of serum M-protein.

		Group A	Group B	Total	p-Value
		Number of patients, %	Number of patients, %		
Sex	Male	54 (57 %)	22 (59 %)	76	0.471
	Female	41 (43 %)	15 (41 %)	56	
Type of heavy chain	G	73 (77 %)	22 (59 %)	95	**0.032**
	A	10 (11 %)	11 (30 %)	21	
	M	12 (12 %)	4 (11 %)	16	
Type of light chain	K	58 (61 %)	20 (54 %)	78	0.294
	L	37 (39 %)	17 (46 %)	54	
Total		95 (72 %)	37 (28 %)	132	

Statistically significant p-values are highlighted in bold. A, alpha; G, gamma; K, kappa; L, lambda; M, mu.

Qualitative variant analysis revealed no statistically significant differences in the distribution of sex and type of light chain between both groups. However, the evaluation of the type of heavy light chain distribution was found to be statistically significant. For this reason, the distribution of IgG, IgA, and IgM between both groups was studied, revealing a higher probability of having M-protein in urine when the isotype was alpha and a lower probability when the isotype was gamma (Table 2).

For those variables with a p-value below 0.05, ROC curves were elaborated (Supplementary Material). These were studied to find cut-offs that yielded optimal predictive values in relation to urine M-protein absence. We observed that when an M-protein concentration in serum of ≤3.5 g/L was used as a cut-off value, 86 % sensitivity and 53 % specificity was obtained, with a 91 % negative predictive value (NPV). When reviewing patients with a serum M-protein ≤3.5 g/L and presence of urine M-protein we stated that they had very low eGFR. For this reason, we added an eGFR >30 mL/min/1.73 m^2^ to the previous cut-off value and with this sensitivity raised to 100 %, with a 49 % specificity and a 100 % NPV of finding M-protein in urine (Table 3). This cut-off would lead us to avoid performing 47 urine analysis (35.6 %) in our study group patients.

**Table 3:** Sensitivities, specificities, and negative predictive values of the proposed cut-off values.

	n	S	Sp	NPV
Serum M-protein ≤3.5 g/L	54	86 %	53 %	91 %
Serum M-protein ≤3.5 g/L+eGFR >30 mL/m/1.73	47	100 %	49 %	100 %
Serum IgG or IgM M-protein ≤4.9 g/L+eGFR >30 mL/m/1.73	56	97 %	52 %	98 %

eGFR, estimated Glomerular Filtration Rate; IgM, immunoglobulin M; IgG, immunoglobulin G; n, number of subjects; NPV, negative predictive value; S, sensitivity; Sp, specificity.

Furthermore, a statistically significant relationship was found between the presence of M-protein in urine and the M-protein heavy chain isotype. Patients with alpha heavy chain had a higher probability of having M-protein in urine (Table 2). Thus, when the heavy chain of the M-protein was gamma or mu, raising the serum cut-off to ≤4.9 g/L and maintaining the eGFR >30 mL/min/1.73 m^2^ led to similar sensitivity (97 %), specificity (52 %), and NPV (98 %) rates (Table 3). In this case 56 urine analysis would have been avoided.

## Discussion

Urine analysis, consisting of UPE and u-IFE, remain an integral part of the comprehensive evaluation when a monoclonal gammopathy is detected in the clinical practice. However, there are discrepancies in clinical guidelines regarding the necessity of these tests, assuming that they may be omitted in certain cases, given that serum techniques can offer sufficient information for the initial diagnosis of these conditions. In the 2014 IMWG guidelines urine analysis does not appear as a diagnostic criterion for MGUS [1]. On the other hand, in the new consensus recommendations for the study of monoclonal gammopathies from the Spanish Society of Laboratory Medicine and the Spanish Society of Haematology and Hemotherapy, urine analysis is not recommended in the screening for monoclonal gammopathies, but it is recommended when a M-protein in serum is detected to complete the study [6].

The role of urine analysis in the diagnosis of monoclonal gammopathies has been studied by several authors. Beetham et al. evaluated the need to include urine studies in the diagnostic algorithm of monoclonal gammopathies. Their results showed that only one out of 105 diagnoses would have been missed when conducting only serum studies (serum electrophoresis, serum immunofixation, and serum free light chains concentration) [7]. Still, they conclude that urine analysis could not be avoided in the screening algorithm for suspected monoclonal gammopathies. Katzmann et al. published a similar study in which only two out of 428 patients with monoclonal gammopathies would have been misdiagnosed when conducting only serum studies. This author concludes that due to the small increase in sensitivity provided by urine studies, these would not be necessary in the initial study and they could be ordered more selectively [8, 9].

In contrast to other published studies, our study proposes a cut-off point from which the urine study could be avoided, with very high NPV (100 % for M-protein concentration of ≤3.5 g/L and 98 % for IgG or IgM M-protein concentration ≤4.9 g/L).

One advantage of incorporating the cut-off values into the laboratory algorithm would be that urine samples would not be necessary for patients who met the criteria, as the parameters needed are determined in serum (serum M-protein and serum creatinine for eGFR CKD-EPI equation). Also the resolved cut-off values also apply to patients with kidney dysfunction with eGFR >30 mL/min/1.73 m^2^.

Of note is that our study was performed in a specific group of patients (first detection of a serum M-protein); therefore, further studies are required to determine whether these cut-offs could yield good predictive values in other patient groups (e.g., patients with presence of two types of M-protein, light chain paraprotein, patients under treatment, patients in remission).

As a result of this study, we can conclude that in patients with recently discovered serum M-protein, urine analysis is not applicable when the concentration of serum M-protein is ≤3.5 g/L and eGFR is >30 mL/min/1.73 m^2^, because the probability of finding M-protein in urine is negligible. In this situation, a significant number of urine analysis could be avoided, thereby optimizing laboratory and healthcare resources.

## Supplementary Material

Supplementary Material
