# Supplementary figures and images for: Urine analysis in monoclonal gammopathies at diagnosis: settling cut-off values
Source: Adv Lab Med. 2024 Sep 23;5(4):439–42. doi: 10.1515/almed-2024-0045 (PMC11661531; doi:10.1515/almed-2024-0045)

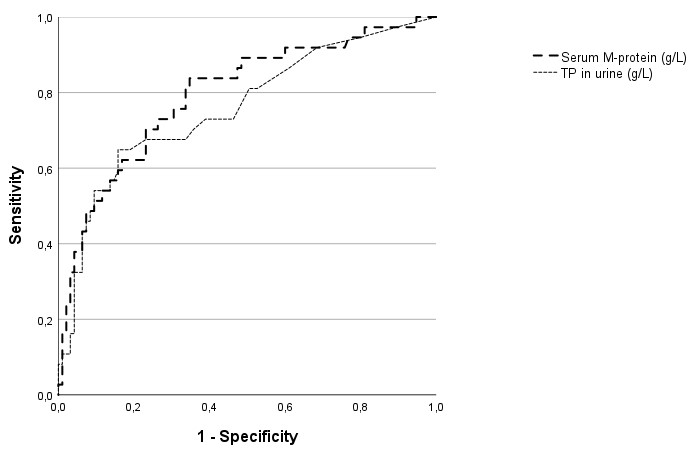
ROC curves


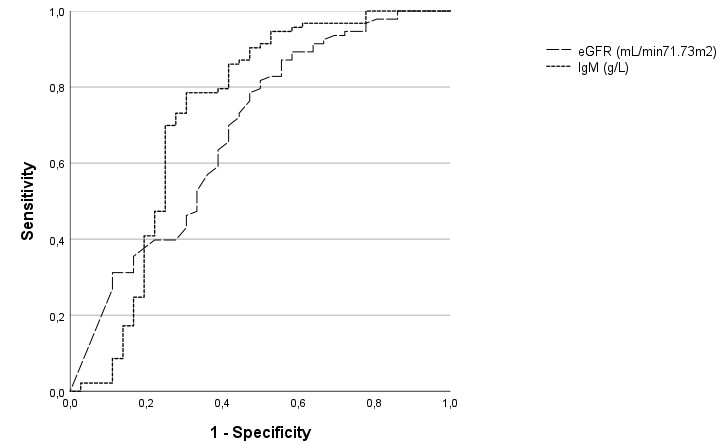

Supplement: Supplementary file 1 — Supplementary Material [file j_almed-2024-0045_suppl_001.docx]
